# The association of lesion eccentricity with plaque morphology and components in the superficial femoral artery: a high-spatial-resolution, multi-contrast weighted CMR study

**DOI:** 10.1186/1532-429X-12-37

**Published:** 2010-07-01

**Authors:** Feiyu Li, Mary McGrae McDermott, Debiao Li, Timothy J Carroll, Daniel S Hippe, Christopher M Kramer, Zhaoyang Fan, Xihai Zhao, Thomas S Hatsukami, Baocheng Chu, Jinnan Wang, Chun Yuan

**Affiliations:** 1 Department of Radiology, Peking University First Hospital, Beijing, China; 2 Department of Radiology, University of Washington, Seattle, WA, USA; 3 Department of Medicine, Northwestern University Feinberg School of Medicine, Chicago, Il, USA; 4 Department of Radiology, Northwestern University Feinberg School of Medicine, Chicago, Il, USA; 5 Departments of Radiology and Medicine, University of Virginia, Charlottesville, VA, USA; 6 Department of Surgery, University of Washington, Seattle, WA, USA; 7 Clinical Sites Research Program, Philips Research North America, Briarcliff Manor, NY, USA

## Abstract

**Background:**

Atherosclerotic plaque morphology and components are predictors of subsequent cardiovascular events. However, associations of plaque eccentricity with plaque morphology and plaque composition are unclear. This study investigated associations of plaque eccentricity with plaque components and morphology in the proximal superficial femoral artery using cardiovascular magnetic resonance (CMR).

**Methods:**

Twenty-eight subjects with an ankle-brachial index less than 1.00 were examined with 1.5T high-spatial-resolution, multi-contrast weighted CMR. One hundred and eighty diseased locations of the proximal superficial femoral artery (about 40 mm) were analyzed. The eccentric lesion was defined as [(Maximum wall thickness- Minimum wall thickness)/Maximum wall thickness] ≥ 0.5. The arterial morphology and plaque components were measured using semi-automatic image analysis software.

**Results:**

One hundred and fifteen locations were identified as eccentric lesions and sixty-five as concentric lesions. The eccentric lesions had larger wall but similar lumen areas, larger mean and maximum wall thicknesses, and more calcification and lipid rich necrotic core, compared to concentric lesions. For lesions with the same lumen area, the degree of eccentricity was associated with an increased wall area. Eccentricity (dichotomous as eccentric or concentric) was independently correlated with the prevalence of calcification (odds ratio 3.78, 95% CI 1.47-9.70) after adjustment for atherosclerotic risk factors and wall area.

**Conclusions:**

Plaque eccentricity is associated with preserved lumen size and advanced plaque features such as larger plaque burden, more lipid content, and increased calcification in the superficial femoral artery.

## Background

Peripheral arterial disease (PAD), a chronic atherosclerotic occlusive disease in the lower extremities, affects 8 to 12 million Americans [1,2]. Local symptoms include intermittent claudication, skin ulcer and progressive extremity gangrene [3]. The risk of death for PAD patients with claudication, especially from coronary and cerebrovascular events, is approximately 2 times greater than the risk for controls without PAD [2,4]. Despite their increased risk of cardiovascular events, patients with PAD are frequently underdiagnosed and undertreated [1].

Previous studies in various arterial beds have demonstrated that plaque compositions are strongly associated with plaque progression, vulnerability and subsequent symptoms [5-7]. Plaques that show intraplaque hemorrhage (IPH), thinned or ruptured fibrous caps and larger lipid rich necrotic cores (LRNC), are more likely to cause cardiovascular events [5,6]. The lower extremity arterial calcification content, measured by computed tomography, has been demonstrated as a potential marker for PAD severity and high risk of amputation in PAD patients [7].

Besides plaque composition, a number of identified morphological characteristics also provide important prognostic information for atherosclerotic lesions, one of which is lesion eccentricity [8]. A few studies suggested that the patients with cerebrovascular and cardiovascular symptoms trended to present with eccentric plaques rather than concentric lesions [9,10]. In addition, the progression of atherosclerosis disease was identified more common in eccentric lesions as compared to concentric geometry [11].

High resolution multi-contrast cardiovascular magnetic resonance (CMR) is a noninvasive technique capable of providing detailed vessel wall information with high accuracy [12]. A number of studies have used multi-contrast CMR as a reliable tool in the assessment of atherosclerotic lesions in various arterial beds, including the carotid, aorta and coronary [13-15]. One prior study demonstrated the reliability and reproducibility of measurement of atherosclerotic plaque volume in the superficial femoral artery [16]. However, to our knowledge, no prior CMR studies have reported on composition of atherosclerotic plaque in the lower extremity arteries.

In this study, we hypothesize that there is a correlation between plaque eccentricity and plaque morphology and composition. These plaque characteristics are measured using high-spatial-resolution multi-contrast CMR in the superficial femoral artery (SFA).

## Methods

### Study population

From February to July 2008, twenty-eight subjects were randomly selected from among participants in the Walking and Leg Circulation Study (WALCS) III cohort. WALCS III participants were identified from consecutive patients with PAD in the non-invasive vascular laboratories at Northwestern Memorial Hospital (Chicago, IL) or from consecutive patients with a diagnosis of PAD who were seen in the vascular surgery, general internal medicine, geriatric, cardiology, or endocrinology clinics at the Northwestern Medical Faculty Foundation (Chicago, IL).

The inclusion criterion was an ankle-brachial index (ABI) <1.00 at the baseline study visit. Exclusion criteria were contraindications to CMR, recent major surgery or cardiovascular event, inability to walk without a wheelchair, stopping during the six-minute walk test due to shortness of breath or arthritic symptoms, inability to speak English, nursing home residence, lower extremity limb amputation, dementia, life expectancy <12 months, and hepatorenal syndrome with associated renal insufficiency. Participant report was collected to acquire information on comorbidities, using established methods [17]. Comorbidities assessed were history of hypertension, diabetes, and coronary artery disease. Cigarette smoking history was measured by self-report. All participants gave informed written informed consent. The study was approved by the institutional review boards of participating sites.

### CMR protocol

All MR scans were performed at a 1.5T (MAGNETOM Espree, Siemens) platform using four-element phased-array surface coils. A standardized protocol (Table 1) was used to obtain 2D bright blood time-of-flight (TOF), black blood T1 weighted, T2 weighted and proton-density weighted (PD) images. Dual regional saturation bands (thickness: 50 mm) 10 mm above and below the imaging volume were applied before data acquisition of each slice to suppress the luminal blood signal. Data for each slice were acquired in an interleaved fashion with each repetition time [18]. Fat suppression was applied in all black-blood sequences to improve identification of vessel outer wall boundaries as well as to avoid chemical shift artifacts. The longitudinal coverage of the superficial femoral artery (SFA) was about 40 mm, beginning at the point where the common femoral artery (CFA) bifurcates into the proximal SFA. After acquisition, all raw data underwent zero-filled interpolation to a matrix size of 512 × 512 pixels.

**Table 1:** Basic scan parameters for the femoral artery imaging protocol at 1.5T

Parameters	Scan type			
	
	1	2	3	4
	
	black-blood PDW	black-blood T1W	black-blood T2W	bright-blood TOF
**Sequence**	TSE	TSE	TSE	GRE
**Image mode**	2D	2D	2D	2D
**Scan plane**	Axial	Axial	Axial	Axial
**TR**, msec	2160	620	2160	38
**TE**, msec	5.7	5.7	51	8.67
**FOV**, cm	12 × 12	12 × 12	12 × 12	12 × 12
**Matrix size**	192 × 192	192 × 192	192 × 192	384 × 384
**Resolution**, mm^2^	0.63 × 0.63	0.63 × 0.63	0.63 × 0.63	0.31 × 0.31
**Slice thickness**, mm	3	3	3	3
**slices**	14	14	14	14
**NEX**	10	12	10	2
**Saturation band**	on	on	on	off
**Fat suppression**	Yes	Yes	Yes	No

TR: repetition time; TE: echo time; NEX: number of excitations

### Image analysis

**Figure 1:**
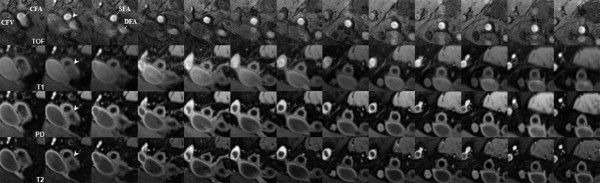
**Example of cross-sectional CMR images with four contrast weightings**. The multi-contrast weighted images were matched using bifurcation (arrowhead) of common femoral artery as a fiducial marker. (CFA: common femoral artery; CFV: common femoral vein; SFA: superficial femoral artery; DFA: deep femoral artery).

The multi-contrast weighted cross-sectional images were matched using the bifurcation of CFA as a fiducial marker (Figure 1). Two trained reviewers (F. L. and X. Z.) with at least one year experience in CMR performed the image review with consensus opinion. Image quality (IQ) was assessed using a previously developed four-point scale (1 = poor quality, 4 = excellent) [19]. Images of occluded SFA or poor image quality were excluded due to unidentifiable lumen and vessel wall. For images with an image quality score of 2 or greater, the lumen area (LA) and total vessel area (TVA) were outlined using semi-automatic image analysis software (CASCADE) [20]. Wall area (WA = TVA-LA), mean/maximum/minimum wall thickness (MeanWT, MaxWT, MinWT) were derived from the LA and TVA outlines.

**Figure 2:**
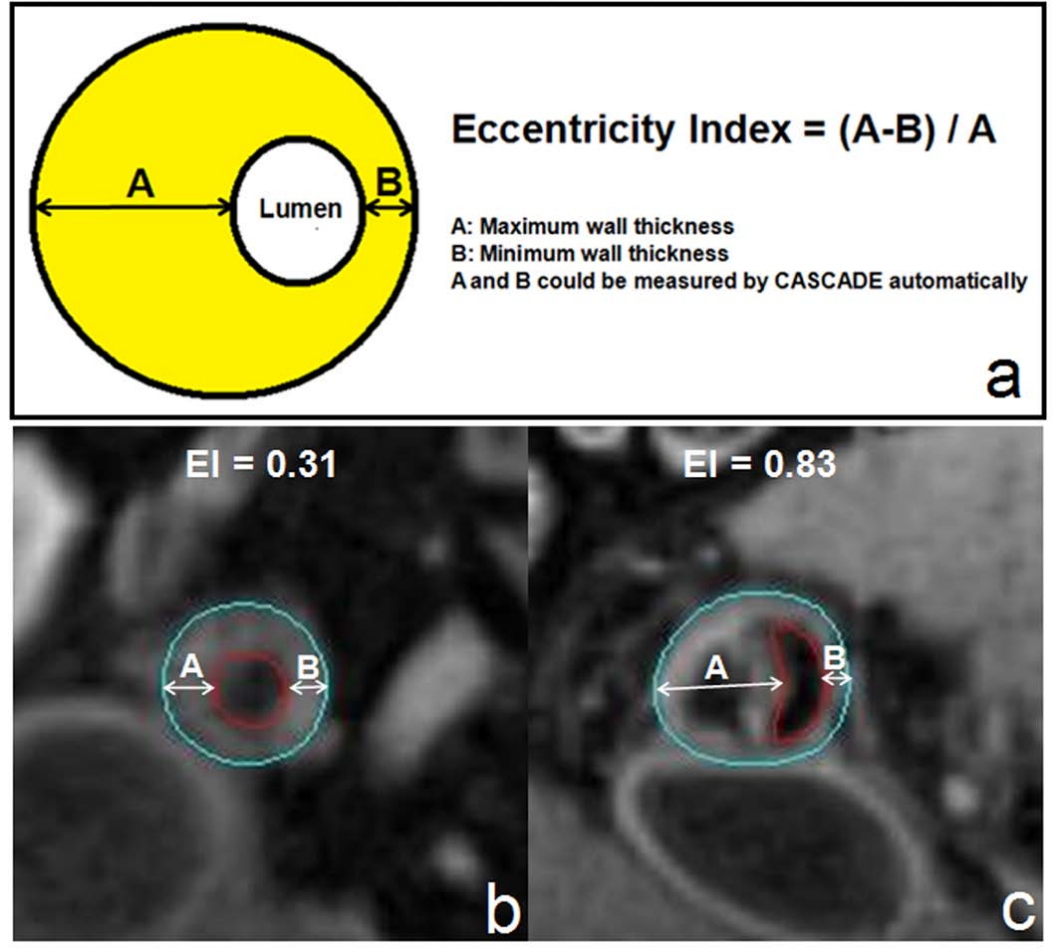
**Delineation of eccentricity measurement**. (a), Eccentricity Index = (A - B)/A. A (Maximum wall thickness) and B (Minimum wall thickness) can be measured by CASCADE automatically. The eccentric lesion is defined as EI ≥ 0.5, concentric lesion as EI < 0.5; (b) and (c), Examples of femoral arterial plaques with eccentricity index of 0.31 and 0.83, respectively. A big calcification can be seen in the eccentric plaque (dark area). (blue: outer wall boundary; red: lumen).

The eccentricity index for each location was calculated by the equation: (MaxWT - MinWT)/MaxWT (Figure 2). A lesion was defined as eccentric if the index was ≥0.5 and as concentric if <0.5 [9].

**Figure 3:**
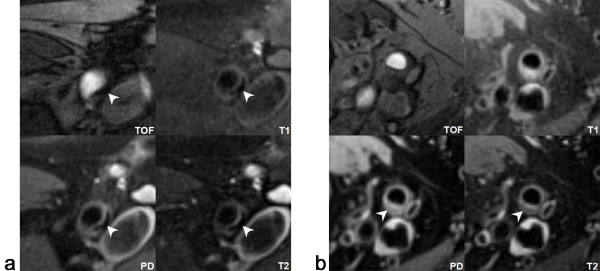
**Example of signal pattern of calcification and lipid rich necrotic core (LRNC) in multi-contrast weighted CMR**. (a) The calcification shows low intensity signal on all four contrast weightings (arrowhead) (b) The typical image pattern of LRNC is iso-intensity on TOF, iso/high-intensity on T1WI and low intensity on PD and T2WI. In the posterior vessel wall of superficial femoral artery, a crescent shaped LRNC can be identified (arrowhead).

Criteria for outlining intraplaque hemorrhage (IPH), lipid rich necrotic core (LRNC) and calcification (CA) were based on previously published carotid CMR criteria for imaging with multi-contrast techniques [12] (Figure 3). Relative content (%) of components was calculated as (component area/wall area)*100.

For each location, lesion type was classified as type I through VIII according to modified American Heart Association (AHA) criteria [13]. Locations with a lesion of type III and above were classified as diseased segments and were analyzed.

### Statistical analysis

Statistical analysis was performed using SPSS for Windows (version 12.0, Chicago, IL) and R version 2.9.2. Quantitative data are presented as mean ± standard deviation (SD). Qualitative data are presented as frequencies.

As the data involved multiple observations from each subject, Generalized Estimating Equation (GEE) models with robust standard error estimators were used to account for intra-subject correlations when performing hypothesis tests [21]. Differences in continuous variables between eccentric and concentric lesions were assessed using contrasts from normal linear regression GEE models. Differences in prevalence of plaque components between eccentric and concentric lesions were assessed using logistic regression GEE models. Except for regression coefficients, all summary statistics (means, standard deviations, frequencies, etc) are presented without any adjustment for intra-subject correlation.

Multivariate GEE logistic regression analysis was performed to correlate the prevalence of plaque components with lesion eccentricity after adjusting for atherosclerosis risk factors (age, gender, hypertension, diabetes, coronary artery disease, smoking and statin therapy) and wall area. All hypothesis tests were conducted within the context of a GEE model were two-sided, and considered significant when the P value was <0.05.

## Results

After exclusion of two subjects with an occluded SFA and twenty-one locations with poor image quality (IQ < 2), 272 cross-sectional SFA images from 26 patients were analyzed. Demographic data for the study population are presented in Table 2.

**Table 2:** Demographic data in study population

Parameter	Datum
**Age (y)**	73.62 ± 8.22
**Height (cm)**	165.73 ± 8.67
**Weight (kg)**	77.53 ± 16. 86
**Male**	54% (14/26)
**History of hypertension**	38% (10/26)
**History of diabetes**	35% (9/26)
**History of coronary artery disease**	46% (12/26)
**Active smoker**	12% (3/26)
**Low density lipoprotein (mg/dl)**	92.38 ± 32.56
**High density lipoprotein (mg/dl)**	51.46 ± 18.70
**Current statin therapy**	77% (20/26)
**Ankle-Brachial Index**	0.64 ± 0.20

Among the 272 locations, 180 locations had a lesion of type III or above, including 81 type III, 16 type IV-V and 83 type VII. According to the eccentricity index (EI), 115 locations were identified as eccentric lesions (eccentricity index ≥ 0.5) and 65 were concentric lesions. In 18 subjects, eccentric and concentric lesions were coexistent in the same arteries. Two subjects showed only concentric and six showed only eccentric lesions.

**Figure 4:**
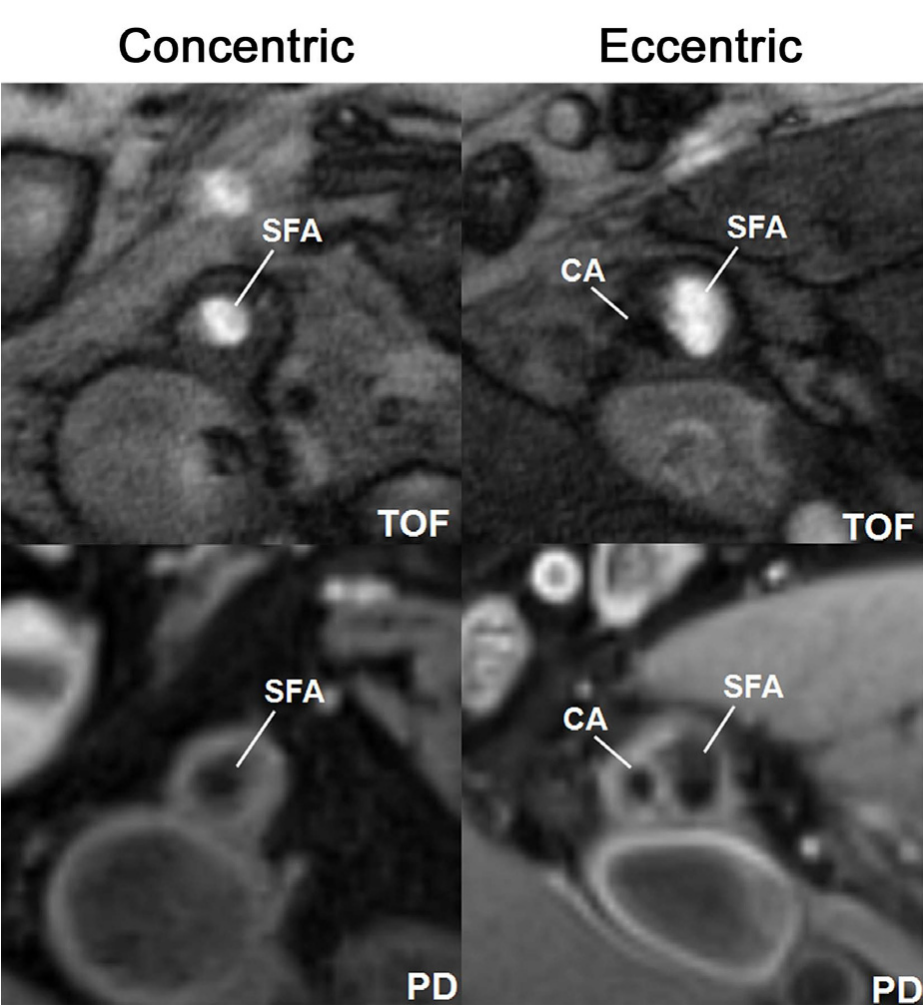
**Example of concentric and eccentric lesions in multi-contrast weighted CMR**. Cross-sectional TOF and PD weighted images show examples of femoral arterial plaques with eccentricity index of 0.39 (concentric) and 0.78 (eccentric), respectively. The eccentric lesion has significant larger wall area (27.50 mm^2 ^vs.15.23 mm^2^), total vessel area (87.65 mm^2 ^vs. 61.55 mm^2^), maximum wall thickness (5.09 mm vs. 2.27 mm) and mean wall thickness (2.43 mm vs. 2.27 mm), but still preserves larger lumen area (27.50 mm^2 ^vs. 15.23 mm^2^). A big piece of calcification (CA), which shows dark signal on all weightings, can be detected in eccentric plaque.

The eccentric lesions exhibited significantly larger wall area, MeanWT, and MaxWT, while there were no significant differences in lumen area. Concentric lesions had larger MinWT (Table 3, Figure 4).

**Table 3:** Comparison of plaque morphology and components between eccentric and concentric lesions

	Eccentricity		
		
	Eccentric	Concentric	P Value
	(mean ± SD)	(mean ± SD)	
**Lumen area, mm**^**2**^	15.3 ± 8.35	13.2 ± 8.8	0.078
**Wall area, mm**^**2**^	38.8 ± 14.9	26.4 ± 10.5	0.019
**Mean wall thickness, mm**	1.98 ± 0.61	1.53 ± 0.31	<0.001
**Max wall thickness, mm**	3.39 ± 1.45	1.94 ± 0.45	<0.001
**Min wall thickness, mm**	1.02 ± 0.19	1.19 ± 0.24	<0.001
**Calcification, mm**^2 ^*	4.90 ± 5.91	1.76 ± 0.55	0.002
% Calcification*	10.1 ± 7.95	5.40 ± 1.34	0.002

* Only locations that exhibited calcification were included; N = 73 for eccentric, N = 11 for concentric.

% Calcification = (calcification area/Wall area) × 100

After adjusting for lumen area in a multivariate linear regression GEE model, eccentricity index was significantly associated with the wall area (p < 0.001). On average, for a given lumen area, each 0.1 increase in eccentricity index was associated with a 9.3% larger wall area.

No intraplaque hemorrhage was detected in these patients. The prevalence of LRNC in eccentric and concentric lesions was 13.0% (15/115) vs. 1.54% (1/65) (P = 0.057). Differences in LRNC size were not considered because there was only a single concentric lesion with a LRNC. For calcification, the prevalence was 63.5% (73/115) in eccentric cross-sections vs. 16.9% (11/65) in concentric cross-sections (P < 0.001). Considering only locations with calcification, eccentric lesions had more calcifications, both in terms of area and relative area (p = 0.002). Using multivariate logistic regression GEE models, the eccentricity (dichotomous as eccentric or concentric) was independently correlated to the presence of calcification (odds ratio 3.78, 95% CI 1.47-9.70) after adjusting for atherosclerotic risk factors and wall area.

## Discussion

The femoral artery hardly tapers and has no major side branches [22], and the atherosclerotic femoral artery could present very diffuse lesions along the whole artery with different eccentricity geometries, which make the femoral artery ideal for the investigation of lesion eccentricity by cross-sectional CMR.

To our knowledge, this is the first study to investigate the association between plaque eccentricity, morphology and components in femoral artery using high-spatial-resolution multi-contrast weighted CMR. Our results demonstrate that plaque composition and morphology are strongly associated with the magnitude of lesion eccentricity.

Pathophysiologic studies described eccentric geometry as a preservation mechanism to maintain the opening of lumen in the presence of atherosclerosis. Hemodynamic forces acting on the endothelium cells are important in the initiation of the atherosclerotic process and pro-atherogenic shear stress profiles produce different plaque phenotypes [23,24]. Low shear stress aggravates eccentric plaque build up, whereas oscillatory shear stress trends to induce small plaques with a concentric phenotype [24]. As a feedback-control loop, localized eccentric plaque distribution also contributes to normal lumen preservation [24], and, by consequence, a low shear stress distribution.

Lumen size measured by angiography has been applied as the gold standard for the evaluation of disease severity and pre-procedural risk assessment before interventions in coronary, carotid and femoral arterial diseases [25]. However, a number of necropsy studies have suggested that plaque size and vulnerability, other than stenosis, may also be important prognostic predictors for cardiovascular events [26,27]. In this study, the bigger plaques with unstable features, such as LRNC, exhibited lumen areas similar to the less advanced lesions due to the occurrence of eccentricity. Thus, a comprehensive assessment of lesion severity in atherosclerotic vessels may include not only the arterial stenosis but also the wall information.

When we controlled for lumen area, the eccentricity index was strongly correlated with wall area. For a fixed lumen area, a 0.1 increase in eccentricity index was associated with a 9.3% increase in wall area. Furthermore, the eccentric lesions trended to contain more LRNC (13.0% vs. 1.54% in concentric), although this finding did not achieve statistical significance (P = 0.057). Our results demonstrate that the prevalence of LRNC is greater in eccentric lesions, compared to concentric lesions. This finding is in accordance with a previous clinical study regarding plaque eccentricity using intravascular ultrasound (IVUS) as image modality in coronary artery [9].

A number of histopathological studies have suggested that LRNC is a crucial determinant of the vulnerability of atherosclerotic lesions [28]. Accumulation of lipid content in eccentric lesion indicates this subgroup may be more likely to rupture and cause subsequent clinical events. T. Ohara, et al. [10] reported that, in patients with a carotid stenosis > 70%, eccentric plaques were associated with a significantly increased incidence of ipsilateral stroke or transient ischemic attack compared to patients with concentric plaques, even though the degrees of arterial stenosis were identical. In a retrospective clinical study including 110 angina patients, Ambrose, et al. [29] found that eccentric lesions were more common in unstable angina than concentric lesions.

Although the clinical significances of calcification in coronary and carotid atherosclerosis are still controversial [30], the calcification in lower extremity arteries has been demonstrated to be strongly associated with more advanced PAD [31]. Guzman et al. [7] reported that calcification content in the tibial artery had the potency to be a better predictor of near-term risk of amputation than a low ABI in patients with PAD. In this study, after adjustment for the atherosclerotic risk factors and wall area in the multivariate logistic regression analysis, eccentricity was independently correlated to prevalence of calcification (odds ratio 3.78). The greater degree of calcification in eccentric lesion implies that the atherosclerotic femoral arteries involved with more eccentric lesions may be related to a worse prognosis than the arteries dominated by concentric lesions despite similar stenosis.

## Limitations

Due to the insufficiency of surface coil coverage, only the cross-sectional MR images in proximal segments (about 40 mm) of SFA were acquired and analyzed in this study. The lesions localized in this segment may not represent the overall plaque characteristics of the entire SFA. A 3D CMR sequence with larger coverage and suitable surface coil may provide more comprehensive imaging than the methods used in the current study. However, the methods reported here are state-of-the-art methods for measuring atherosclerotic plaque in the femoral arteries, and further work is necessary before 3D imaging can be used successfully to image atherosclerotic plaques in the superficial femoral artery in PAD patients. In addition, the sample size was small in this study. Further studies in a larger population are needed before these results can be generalized to the population at large.

## Conclusions

The eccentricity of atherosclerotic plaque in the superficial femoral artery preserves lumen size and is associated with advanced plaque features in vessel wall, such as larger plaque burden, more lipid content and increased calcification. Further longitudinal study is needed to identify the prognostic significance of plaque eccentricity in the superficial femoral artery.

## Competing interests

The authors declare that they have no competing interests.

## Authors' contributions

FL conceived and designed the study, participated the data analysis and draft of the manuscript. MMM obtained funding, was involved in the study design of non-CMR data collection methods, provided oversight to all aspects of non-CMR data collection, and oversaw subject recruitment. DH gave a major contribution to the data. DL and TJC were responsible for obtaining funding, the CMR protocol and image acquisition. XH was involved in the image analysis. CMK, TSH and JW helped in coordinating the study and contributed to the manuscript draft. YC participated in the study design, assisted with obtaining funding, data analysis and the revision of the manuscript. All authors read and approved the final manuscript.

## References

[B1] Hirsch AT Criqui MH Treat-Jacobson D Regensteiner JG Creager MA Olin JW Krook SH Hunninghake DB Comerota AJ Walsh ME McDermott MM Hiatt WR Peripheral arterial disease detection, awareness, and treatment in primary care JAMA 2001 286 1317 1324 10.1001/jama.286.11.1317 11560536

[B2] Criqui MH Langer RD Fronek A Feigelson HS Klauber MR McCann TJ Browner D Mortality over a period of 10 years in patients with peripheral arterial disease N Engl J Med 1992 326 381 386 1729621 10.1056/NEJM199202063260605

[B3] Cimminiello C PAD. Epidemiology and pathophysiology Thromb Res 2002 106 V295 301 10.1016/S0049-3848(01)00400-5 12359342

[B4] Lumsden AB Rice TW Chen C Zhou W Lin PH Bray P Morrisett J Nambi V Ballantyne C Peripheral arterial occlusive disease: magnetic resonance imaging and the role of aggressive medical management World J Surg 2007 31 695 704 10.1007/s00268-006-0732-y 17345122

[B5] Takaya N Yuan C Chu B Saam T Underhill H Cai J Tran N Polissar NL Isaac C Ferguson MS Garden GA Cramer SC Maravilla KR Hashimoto B Hatsukami TS Association between carotid plaque characteristics and subsequent ischemic cerebrovascular events: a prospective assessment with MRI--initial results Stroke 2006 37 818 823 10.1161/01.STR.0000204638.91099.91 16469957

[B6] Davies MJ Thomas AC Plaque fissuring--the cause of acute myocardial infarction, sudden ischaemic death, and crescendo angina Br Heart J 1985 53 363 373 10.1136/hrt.53.4.363 3885978 PMC481773

[B7] Guzman RJ Brinkley DM Schumacher PM Donahue RM Beavers H Qin X Tibial artery calcification as a marker of amputation risk in patients with peripheral arterial disease J Am Coll Cardiol 2008 51 1967 1974 10.1016/j.jacc.2007.12.058 18482666 PMC2836514

[B8] Mintz GS Popma JJ Pichard AD Kent KM Satler LF Chuang YC DeFalco RA Leon MB Limitations of angiography in the assessment of plaque distribution in coronary artery disease: a systematic study of target lesion eccentricity in 1446 lesions Circulation 1996 93 924 931 8598083 10.1161/01.cir.93.5.924

[B9] Yamagishi M Terashima M Awano K Kijima M Nakatani S Daikoku S Ito K Yasumura Y Miyatake K Morphology of vulnerable coronary plaque: insights from follow-up of patients examined by intravascular ultrasound before an acute coronary syndrome J Am Coll Cardiol 2000 35 106 111 10.1016/S0735-1097(99)00533-1 10636267

[B10] Ohara T Toyoda K Otsubo R Nagatsuka K Kubota Y Yasaka M Naritomi H Minematsu K Eccentric stenosis of the carotid artery associated with ipsilateral cerebrovascular events AJNR Am J Neuroradiol 2008 29 1200 1203 10.3174/ajnr.A0997 18339721 PMC8118845

[B11] Nair P Gruberg L Beyar R The eccentric lumenology Acute Card Care 2006 8 87 94 10.1080/17482940600765950 16885072

[B12] Saam T Ferguson MS Yarnykh VL Takaya N Xu D Polissar NL Hatsukami TS Yuan C Quantitative evaluation of carotid plaque composition by in vivo MRI Arterioscler Thromb Vasc Biol 2005 25 234 239 10.1161/01.ATV.0000155965.54679.79 15528475

[B13] Cai JM Hatsukami TS Ferguson MS Small R Polissar NL Yuan C Classification of human carotid atherosclerotic lesions with in vivo multicontrast magnetic resonance imaging Circulation 2002 106 1368 1373 10.1161/01.CIR.0000028591.44554.F9 12221054

[B14] Kramer CM Cerilli LA Hagspiel K DiMaria JM Epstein FH Kern JA Magnetic resonance imaging identifies the fibrous cap in atherosclerotic abdominal aortic aneurysm Circulation 2004 109 1016 1021 10.1161/01.CIR.0000116767.95046.C2 14967731 PMC2957882

[B15] Sun B Giddens DP Long R Jr Taylor WR Weiss D Joseph G Vega D Oshinski JN Characterization of coronary atherosclerotic plaque using multicontrast MRI acquired under simulated in vivo conditions J Magn Reson Imaging 2006 24 833 841 10.1002/jmri.20687 16929530

[B16] Isbell DC Meyer CH Rogers WJ Epstein FH DiMaria JM Harthun NL Wang H Kramer CM Reproducibility and reliability of atherosclerotic plaque volume measurements in peripheral arterial disease with cardiovascular magnetic resonance J Cardiovasc Magn Reson 2007 9 71 76 10.1080/10976640600843330 17178683 PMC2927819

[B17] McDermott MM Ades P Guralnik JM Dyer A Ferrucci L Liu K Nelson M Lloyd-Jones D Van Horn L Garside D Kibbe M Domanchuk K Stein JH Liao Y Tao H Green D Pearce WH Schneider JR McPherson D Laing ST McCarthy WJ Shroff A Criqui MH Treadmill exercise and resistance training in patients with peripheral arterial disease with and without intermittent claudication: a randomized controlled trial JAMA 2009 301 165 174 10.1001/jama.2008.962 19141764 PMC3268032

[B18] Koktzoglou I Chung YC Mani V Carroll TJ Morasch MD Mizsei G Simonetti OP Fayad ZA Li D Multislice dark-blood carotid artery wall imaging: a 1.5 T and 3.0 T comparison J Magn Reson Imaging 2006 23 699 705 10.1002/jmri.20563 16555260

[B19] Underhill HR Yarnykh VL Hatsukami TS Wang J Balu N Hayes CE Oikawa M Yu W Xu D Chu B Carotid plaque morphology and composition: initial comparison between 1.5- and 3.0-T magnetic field strengths Radiology 2008 248 550 560 10.1148/radiol.2482071114 18574135 PMC2797646

[B20] Kerwin W Xu D Liu F Saam T Underhill H Takaya N Chu B Hatsukami T Yuan C Magnetic resonance imaging of carotid atherosclerosis: plaque analysis Top Magn Reson Imaging 2007 18 371 378 10.1097/rmr.0b013e3181598d9d 18025991

[B21] Zeger SL Liang KY Longitudinal data analysis for discrete and continuous outcomes Biometrics 1986 42 121 130 10.2307/2531248 3719049

[B22] Vink A Schoneveld AH Richard W de Kleijn DP Falk E Borst C Pasterkamp G Plaque burden, arterial remodeling and plaque vulnerability: determined by systemic factors? J Am Coll Cardiol 2001 38 718 723 10.1016/S0735-1097(01)01444-9 11527623

[B23] Davies PF Mechanisms involved in endothelial responses to hemodynamic forces Atherosclerosis 1997 131 Suppl S15 17 10.1016/S0021-9150(97)06118-2 9253470

[B24] Helderman F Segers D de Crom R Hierck BP Poelmann RE Evans PC Krams R Effect of shear stress on vascular inflammation and plaque development Curr Opin Lipidol 2007 18 527 533 10.1097/MOL.0b013e3282ef7716 17885423

[B25] Just H Coronary arteriography: current technique and standards of equipment Med Prog Technol 1977 5 119 125 340873

[B26] Kullo IJ Edwards WD Schwartz RS Vulnerable plaque: pathobiology and clinical implications Ann Intern Med 1998 129 1050 1060 9867761 10.7326/0003-4819-129-12-199812150-00010

[B27] Shin J Edelberg JE Hong MK Vulnerable atherosclerotic plaque: clinical implications Curr Vasc Pharmacol 2003 1 183 204 10.2174/1570161033476727 15320843

[B28] Virmani R Burke AP Farb A Kolodgie FD Pathology of the vulnerable plaque J Am Coll Cardiol 2006 47 C13 18 10.1016/j.jacc.2005.10.065 16631505

[B29] Ambrose JA Winters SL Stern A Eng A Teichholz LE Gorlin R Fuster V Angiographic morphology and the pathogenesis of unstable angina pectoris J Am Coll Cardiol 1985 5 609 616 10.1016/S0735-1097(85)80384-3 3973257

[B30] Abedin M Tintut Y Demer LL Vascular calcification: mechanisms and clinical ramifications Arterioscler Thromb Vasc Biol 2004 24 1161 1170 10.1161/01.ATV.0000133194.94939.42 15155384

[B31] Niskanen L Siitonen O Suhonen M Uusitupa MI Medial artery calcification predicts cardiovascular mortality in patients with NIDDM Diabetes Care 1994 17 1252 1256 10.2337/diacare.17.11.1252 7821163

